# Surveillance for radiation‐related late effects in childhood cancer survivors: The impact of using volumetric dosimetry

**DOI:** 10.1002/cam4.3671

**Published:** 2020-12-16

**Authors:** Sally Cohen‐Cutler, Arthur Olch, Kenneth Wong, Jemily Malvar, Richard Sposto, Pierre Kobierski, Amit Sura, Louis S. Constine, David R. Freyer

**Affiliations:** ^1^ Cancer and Blood Disease Institute Children's Hospital Los Angeles Los Angeles CA USA; ^2^ Radiation Oncology Program Children's Hospital Los Angeles Los Angeles CA USA; ^3^ Keck School of Medicine University of Southern California Los Angeles CA USA; ^4^ Department of Preventive Medicine Keck School of Medicine University of Southern California Los Angeles CA USA; ^5^ Department of Radiology Children's Hospital Los Angeles Los Angeles CA USA; ^6^ Departments of Radiation Oncology and Pediatrics James P Wilmot Cancer Institute University of Rochester Medical Center Rochester NY USA; ^7^ Departments of Pediatrics and Medicine Keck School of Medicine University of Southern California Los Angeles CA USA

**Keywords:** cancer survivors, child, delivery of health care/methods, organs at risk, radiotherapy

## Abstract

**Background:**

Radiation‐related screening guidelines for survivors of childhood cancer currently use irradiated regions (IR) to determine risk for late effects. However, contemporary radiotherapy techniques utilize volumetric dosimetry (VD) to determine organ‐specific exposures, which could inform need for late effect surveillance.

**Methods:**

This cross‐sectional cohort study involved patients treated for cancer using computerized tomography‐planned irradiation at Children's Hospital Los Angeles from 2000–2016. Organs at risk were identified using both VD and IR. Under each method, Children's Oncology Group Long‐Term Follow‐Up Guidelines were applied to determine radiation‐related potential late effects and their correlative recommended screening practices. Patients served as their own controls. Mean number of potential late effects per patient and recommended screening practices per patient per decade of follow‐up were compared using paired t‐tests; comparisons were adjusted for diagnosis and gender using random effects, repeated measure linear regression.

**Results:**

In this cohort (n = 132), median age at end of treatment was 10.6 years (range, 1.4–20.4). Brain tumor was the most common diagnosis (45%) and head/brain the most common irradiated region (61%). Under IR and VD, the mean number of potential late effects flagged was 24.4 and 21.7, respectively (−11.3%, *p* < 0.001); concordance between the two methods was 6.1%. Under VD, the difference in mean number of recommended screening practices per patient was −7.4% in aggregate but as large as −37.0% for diagnostic imaging and procedures (*p* < 0.001 for both).

**Conclusion:**

Use of VD rather than IR is feasible and enhances precision of guideline‐based screening for radiation‐related late effects in long‐term childhood cancer survivors.

## Graphical abstract

In this cross‐sectional cohort study of 132 eligible childhood cancer survivors treated with radiation therapy, we utilized current Children's Oncology Group Long‐term Follow‐Up Guidelines to compare the recommended screening practices triggered by use of conventional radiation fields ("irradiated regions") versus the more precise approach of volumetric dosimetry. This figure shows the number of recommended screening practices per patient per decade of follow‐up, by volumetric dosimetry (VD) compared with irradiated regions (IR). Each scatter plot point represents one patient with the number of recommended screening practices as determined by IR (x‐axis) versus VD (y‐axis); patients below the dotted line had fewer screening practices triggered using VD while those above it had more. Results are shown for all patients (A); by diagnosis (BSTT=bone/soft tissue tumor, LL=leukemia and lymphoma, CNS=central nervous system tumor) (B); by gender (C); and by age at end of therapy (D) Under IR and VD, the mean number of potential late effects flagged was 24.4 and 21.7, respectively (−11.3%, *p* < 0.001); full concordance between the two methods was 6.1%. We found that under VD, the difference in mean number of recommended screening practices per patient was −7.4% in aggregate but as large as −37.0% for diagnostic imaging and procedures (*p* < 0.001 for both). Study results show that use of VD rather than IR is feasible and enhances precision of guideline‐based screening for radiation‐related late effects in long‐term childhood cancer survivors.

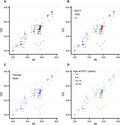

## 1 INTRODUCTION

Treatment advances have greatly improved outcomes for children and adolescents diagnosed with cancer, such that 5‐year survival now exceeds 85%.^1^, ^2^ However, most long‐term survivors develop late effects caused by cancer treatment.^1^ Radiation therapy causes many predictable and clinically significant late effects, including second malignancies, neurocognitive deficits, cardiotoxicity, cerebrovascular disease, and musculoskeletal deformity, often compromising health and quality of life.^1^, ^3^, ^4^, ^5^ Because early detection may mitigate these complications, regular late effects screening and health promotion are recommended as best practice in survivorship care.^1^, ^3^, ^6^

In North America, late effects surveillance is based upon the Children's Oncology Group (COG) Long‐Term Follow‐Up Guidelines for Survivors of Childhood, Adolescent, and Young Adult Cancer.^7^ Introduced in 2004, the COG guidelines utilize published evidence and expert consensus in recommending late effects screening practices and are updated regularly.^8^ The recommendations are organized by treatment modality (chemotherapy, surgery, and/or radiation); the type, frequency, and duration of screening measures are determined by individual risk. For survivors treated with radiation therapy, recommendations are determined by radiation fields encompassing organs at risk (OAR) that are screened based on presumed radiation exposure. Use of radiation fields, which we designate herein irradiated regions (IR), offers the advantage of ready applicability to clinical practice, but provides limited, potentially inaccurate, estimates about true exposure of any OAR within or adjacent to these regions.

In contrast, current radiotherapy techniques utilize three‐dimensional treatment planning to determine organ‐specific dose‐volume data, i.e., volumetric dosimetry (VD).^9^, ^10^, ^11^ Compared with IR, VD could be expected to estimate more accurately organ‐specific radiation exposure and need for late effects screening.^11^, ^12^ However, no studies have been published, to our knowledge, evaluating the direct impact of incorporating VD into guideline‐based late effects surveillance. Such an effort would be responsive to the recent call for greater “precision survivorship.”^13^

To explore this possibility, we undertook this cohort study to evaluate the feasibility and impact of using VD for determining recommended radiation‐related late effects screening in childhood cancer survivors. The primary aim was to compare, by using VD versus IR, the potential late effects identified, and type and frequency of screening practices recommended by the COG guidelines. Our hypothesis was that use of VD would more precisely identify at risk organs and consequential potential late effects, resulting in a net decrease in recommended screening practices.

## 2 METHODS

### 2.1 Study design and participants

This was a cross‐sectional cohort study of childhood cancer survivors previously treated with radiation therapy at Children's Hospital Los Angeles (CHLA) and identified using our LIFE Cancer Survivorship Research Database. The LIFE Database contains patient‐specific demographics and information on cancer diagnosis and treatment. The LIFE Database is approved by the CHLA Institutional Review Board (IRB); informed consent/assent is prospectively obtained prior to registration in the database.

Inclusion criteria included exclusively computerized tomography (CT)‐planned radiotherapy given for a first diagnosis of cancer at CHLA between 2000 (when volumetric CT planning became standard at CHLA) and 2016. Cancer diagnoses were divided into Bone/Soft Tissue Tumors (BSTT), Leukemia and Lymphoma (LL), and Central Nervous System Tumors (CNS). This study was approved by the CHLA IRB.

### 2.2 Late effect screening guidelines

Late effects and recommended screening practices referenced in this study were drawn from the COG guidelines (version 5.0), which contains 55 sections describing potential radiation‐related late effects in OAR (Appendix Table A1) and their corresponding periodic evaluations and health counseling.^7^

### 2.3 Determination of organ radiation exposures

For each patient, radiation exposure was determined by two methods. To determine exposure by irradiated regions (IR), we referred to radiation fields as defined by the COG guidelines: head/brain, neck, axilla, chest, abdomen, pelvis, testicular, cervical, thoracic, lumbar, and sacral spine, as well as total body irradiation. These were then used to determine OAR as specified by COG guidelines. To determine organ‐specific volumetric dosimetry (VD), organs visible on the radiotherapy planning CT scan were contoured using treatment planning software (Eclipse, Varian Medical Systems). OAR for approximately 30% of patients were drawn manually. Representative patients were used to create a reference organ contour atlas in an auto‐segmentation program (Velocity, Varian Medical Systems) that was applied to remaining patients. For quality assurance, all contoured structures were reviewed by an experienced radiologist (A.S.) and edited as needed.

Organ‐specific volumetric dose calculations performed by the Eclipse system were then transferred into the LIFE Database using the LIFE Automated Dose‐Volume Retrieval System (LADDRS), a web‐based computer program developed at CHLA using an application program interface script for extracting data from the Eclipse treatment planning system and Microsoft Structured Query Language for data from the Aria database (Varian Medical Systems). This program exports organ‐specific volumetric doses and, using predetermined limits, flags potentially toxic doses.^14^

For purposes of this study, VD limits matched toxicity thresholds set by the COG guidelines for OAR and corresponding potential late effects. Only five OAR have specific radiation dose thresholds defined by COG guidelines: hypothalamic‐pituitary axis (30 Gray [Gy]), cochlea (30 Gy), mandible (40 Gy), heart (15 Gy), and spleen (40 Gy). For these, the threshold to trigger screening by VD was set as 50% of the contoured organ having received the guideline‐specified dose. For the remaining 50 OAR without dose limits specified by the COG guidelines, our VD‐determined screening threshold was set as 10% of the organ receiving a minimum of 5 Gy. For bilateral organs, late effect screening was triggered if either one exceeded the threshold.

### 2.4 Study procedures and outcome variables

For each patient, radiation exposures were determined by the two methods, IR and VD, as described above. Next, the COG guidelines were applied to generate and compare, as derived by each method, the (1) potential late effects that could be incurred; and (2) corresponding recommended screening practices. Thus, patients served as their own controls. For study purposes, recommended screening practices were further categorized as *history and physical examination elements*, *laboratory tests*, *diagnostic imaging and procedures*, *referrals to specialists*, and *health education and counseling* (Appendix Table A2). In applying the COG guidelines, standard demographic and relevant treatment factors were considered, including ages at treatment and screening, sex, maximum radiation dose in regions involving the heart, and cumulative anthracycline exposure due to its incorporation in determining radiation‐related risk. End of puberty was set at 16 years for females and 18 years for males. To estimate lifetime screening burden, recommendations were summed over an assumed remaining lifespan until 80 years of age. When a screening practice was triggered by multiple organ exposures, only the one with greatest screening frequency was used. Data were managed using REDCap.^15^, ^16^

### 2.5 Statistical analysis

Descriptive statistics were used for the number and type of potential late effects and recommended screening practices according to each method of determining radiation exposure (IR and VD). Mean number of potential late effects was computed for each approach and compared using paired t‐tests. Mean number of recommended screening practices per patient per decade of follow‐up was determined for each approach and was compared using paired t‐tests. Random effects, repeated measures linear regression was utilized to assess differences in recommended screening practices while controlling for effects of gender, age at end of treatment, and cancer type. For each of the five potential late effects that have dose‐specific thresholds assigned by COG guidelines, the number of patients at risk was determined by IR and VD; corresponding recommended screening practices were then calculated by each method over the remaining lifespan and compared. All analyses utilized two‐sided tests with significance set at *p* < 0.05 and were completed using Stata statistical software.^17^

## 3 RESULTS

### 3.1 Patient characteristics

A total of 509 patients who received radiation therapy were identified. After excluding those who were treated before 2000 (n = 160), did not undergo complete CT planning (n = 150), or had relapsed (n = 67), 132 were included. Key clinical and treatment characteristics for the cohort are shown in Table 1. Median ages at diagnosis and end of therapy were 9.7 and 10.6 years respectively. Over 80% of patients were treated for solid tumors; CNS tumors accounted for almost half (n = 59). Over 80% of patients received radiation to 1–2 unique regions as defined by the COG guidelines; the maximum number of regions was 5 and the mean was 1.8. The three most common irradiated regions were head/brain (81, 61%), abdomen (29, 22%), and spine (20, 15%). Forty‐one percent of patients received anthracyclines with a mean cumulative doxorubicin‐equivalent dose of 237 mg/m^2^.

**TABLE 1:** Study cohort characteristics (n = 132)

Clinical characteristics	
Age (y)	
*At diagnosis*	
Median	9.7
Range	0.3–19.8
*At end of treatment*	
Median	10.6
Range	1.4–20.4
Gender	n (%)
Male	80 (61)
Female	52 (39)
Diagnosis type	n (%)
*Central nervous system tumor*	59 (45)
Germ cell tumor	12 (9)
Ependymoma	12 (9)
Medulloblastoma	12 (9)
Craniopharyngioma	7 (5)
Other^a^	16 (12)
*Bone/soft tissue tumor*	52 (39)
Rhabdomyosarcoma	14 (11)
Neuroblastoma	13 (10)
Wilms tumor	5 (4)
Retinoblastoma	4 (3)
Ewing sarcoma	4 (3)
Other^b^	12 (9)
*Leukemia/lymphoma*	21 (16)
Hodgkin lymphoma	14 (11)
Non‐Hodgkin lymphoma	3 (2)
Leukemia	4 (3)

Radiation exposure	
Unique regions per patient (no.)	n (%)
1	86 (65)
2	21 (16)
3	4 (3)
4	5 (4)
5	16 (12)
Prescribed irradiated regions	
*Head/Brain*	81 (61)
Mean prescribed dose ±SD (Gy)	48.8 ± 13.5
Median prescribed dose (range)	54 (12–66.6)
*Abdomen*	29 (22)
Mean prescribed dose ±SD (Gy)	27.8 ± 12.4
Median prescribed dose (range)	21.6 (9–55.8)

*Chest*	20 (15)
Mean prescribed dose ±SD (Gy)	22.7 ± 13
Median prescribed dose (range)	21 (10.5–66)
*Spine*	20 (15)
Mean prescribed dose ±SD (Gy)	31.1 ± 13.6
Median prescribed dose (range)	23.4 (6–54)
*Neck*	19 (14)
Mean prescribed dose ±SD (Gy)	26.7 ± 10.6
Median prescribed dose (range)	21 (21–51.2)
*Pelvis*	12 (9)
Mean prescribed dose ±SD (Gy)	42.8 ± 14.4
Median prescribed dose (range)	50.4 (21–56)
*Extremity*	10 (8)
Mean prescribed dose ±SD (Gy)	46.7 ± 18.1
Median prescribed dose (range)	50.4 (21.6–70)
*Axilla*	8 (6)
Mean prescribed dose ±SD (Gy)	21 ± 0
Median prescribed dose (range)	21 (21)

Anthracycline exposure	n (%)
Exposed	54 (41)
Cumulative dose (mg/m^2^)^c^	
0	78 (59)
<250	34 (26)
≥250	20 (15)

^a^ Other central nervous system tumors included astrocytoma (n = 4), glioma (n = 3), atypical teratoid rhabdoid tumor (n = 2), primitive neuro‐ectodermal tumors (n = 2), chondrosarcoma (n = 1), choroid plexus carcinoma (n = 1), giant cell glioblastoma (n = 1), gliosarcoma (n = 1), mixed tumor (n = 1)

^b^ Other bone/soft tissue tumors included malignant peripheral nerve sheath tumor (n = 2), nasopharyngeal carcinoma (n = 2), synovial sarcoma (n = 2), acinar cell carcinoma (n = 1), adenoid cystic carcinoma (n = 1), angiofibroma (n = 1), chondrosarcoma (n = 1), clear cell sarcoma (n = 1), pancreatoblastoma (n = 1)

^c^ Anthracycline dose in doxorubicin equivalents^7^

### 3.2 Potential late effects flagged

**FIGURE 1:**
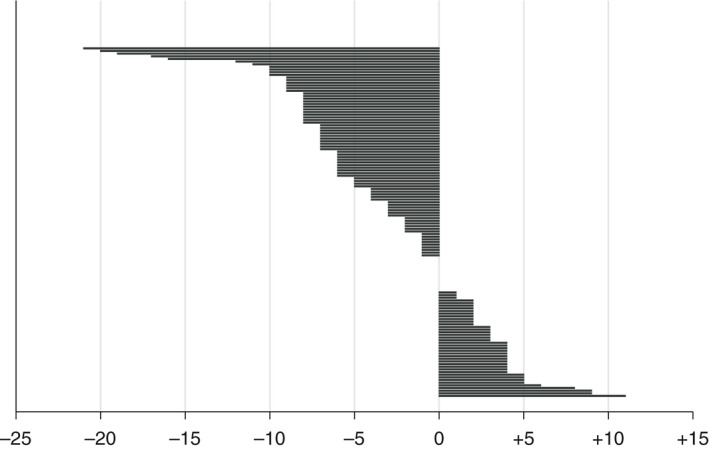
Difference in the number of potential late effects flagged by volumetric dosimetry (VD) compared with irradiated regions (IR), by patient (n = 132). Each line of the waterfall plot represents one patient. A smaller or larger number of potential late effects flagged using VD is quantified on the x‐axis using negative or positive values, respectively; for patients with no corresponding line, there was no difference

We first compared the number and type of potential late effects per patient flagged for surveillance by IR and VD. For the full cohort, the mean number of late effects flagged by IR and VD was 24.4 versus 21.7, respectively (mean difference −2.8 [−11.3%], *p* < 0.001). Seventy‐nine patients (59.9%) had fewer late effects flagged, 40 (30.3%) had more, and 13 (9.8%) had no change (Figure 1). For those with fewer, the mean decrease was −6.5, and for those with more, the mean increase was 3.8. Among the 13 patients with the same number of late effects flagged, eight (6.1%) had exactly the same combination flagged by both methods; four of those had only radiation of an extremity with no potential organ exposure, effectively reducing that number to 4 (3.0%).

### 3.3 Recommended screening practices triggered

**FIGURE 2:**
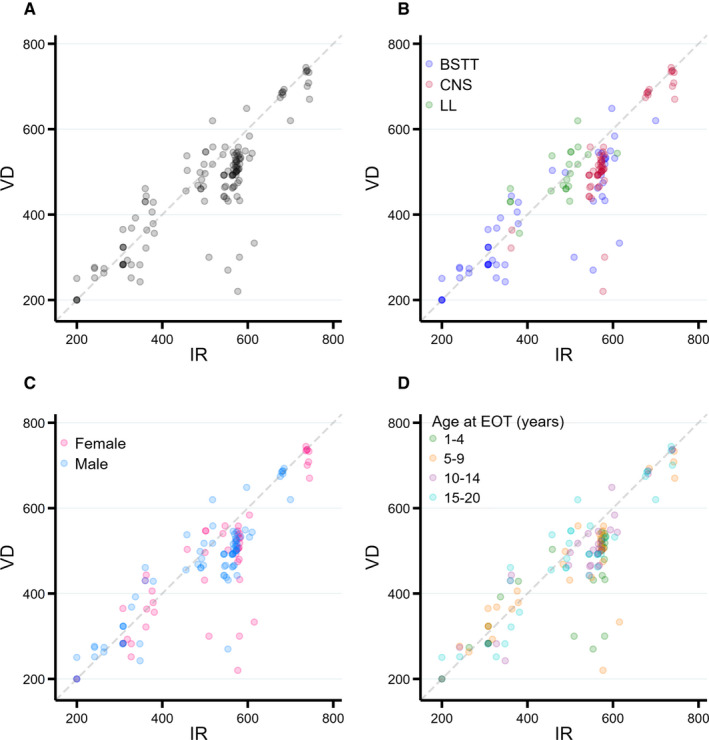
Number of recommended screening practices per patient per decade of follow‐up, by volumetric dosimetry (VD) compared with irradiated regions (IR). Each scatter plot point represents one patient with the number of recommended screening practices as determined by IR (x‐axis) versus VD (y‐axis); patients below the dotted line had fewer screening practices triggered using VD while those above it had more. Results are shown for all patients (A); by diagnosis (BSTT=bone/soft tissue tumor, LL=leukemia and lymphoma, CNS=central nervous system tumor) (B); by gender (C); and by age at end of therapy (D)

Taking all screening practices in aggregate, there was a tendency for patients to have fewer screening practices triggered with VD than with IR, especially for patients needing approximately 500–600 screening practices per decade of follow‐up (Figure 2, Panel A). Using VD, 91 (68.9%) patients had fewer recommended screening practices, 34 (25.8%) had more, and 8 (6.1%) had the same number. This reduction with VD appeared to be more striking for CNS tumors compared with other cancer types (Panel B) but was relatively balanced by gender (Panel C) and age (Panel D).

**FIGURE 3:**
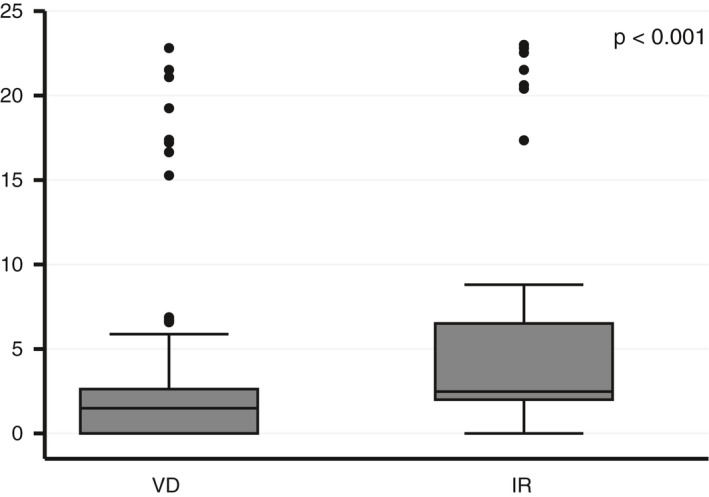
Number of diagnostic imaging and procedures per patient per decade of follow‐up, box plot comparison of screening recommendations triggered using volumetric dosimetry (VD) versus irradiated regions (IR)

To quantify these findings for relevancy to clinical practice, we computed the mean number of recommended screening practices per patient per decade of follow‐up and determined the difference between the two methods, shown in Table 2. With VD, the aggregate number of recommended screening practices triggered was significantly reduced compared with IR (−7.4%, *p* < 0.001). By specific category of screening practice, reductions of similar magnitude using VD were noted for *history and physical examination elements* (−6.9%, *p* < 0.001), *referrals to specialists* (−9.9%, *p* < 0.001), and *health education and counseling* (−7.7%, *p* < 0.001). The most substantial reduction was noted for *diagnostic imaging and procedures*, where 37% fewer tests per patient were triggered with use of VD (Table 2, Figure 3; *p* < 0.001). The number of *laboratory tests* triggered was also lower with VD (−4.6%) but not significantly so.

**TABLE 2:** Number of recommended screening practices per patient per decade of follow‐up

	Irradiated regions		Volumetric dosimetry					
Screening practice	Mean	Range	Mean	Range	Absolute difference^a^	95% CI^a^	% Difference^a^	*p*‐value^a^
All screening practices	504.4	200–744.4	467.2	200–744.4	−37.2	−49.2, −25.2	−7.4%	<0.001
History and physical exam elements	396	180–568.4	368.5	180–560	−27.5	−36.2, −18.8	−6.9%	<0.001
Laboratory tests	15.3	0–20.3	14.6	0–30.3	−0.7	−1.8, 0.5	−4.6%	0.265
Diagnostic imaging and procedures	4.6	0–23	2.9	0–22.8	−1.7	−2.3, −1.1	−37.0%	<0.001
Referrals to specialists	21.2	0–30.2	19.1	0–30.2	−2.1	−3.2, −1.0	−9.9%	<0.001
Health education and counseling	67.4	20–120.2	62.1	20–120.2	−5.2	−8, −2.5	−7.7%	<0.001

^a^ As determined with random effects repeated measures linear regression

In the current version of the COG guidelines, only five organs or anatomic structures have radiation dose‐specific thresholds identified for triggering health screening and counseling (see Methods). The lifetime impact of using IR and VD in these structures is shown in Table 3. Without exception, for every potential late effect and corresponding recommended screening practice, use of VD resulted in a substantial reduction in the absolute number of interventions needed. Of the 15 recommended screening practices, testing for the cohort was reduced by more than half for 9 and more than a third for 13. Using VD almost eliminated testing for two screening practices (evaluation for osteoradionecrosis of mandible and infectious risk counseling for functional asplenia). Somewhat lesser reductions were seen for focused endocrine history and medical alert bracelet counseling (central adrenal insufficiency), and for blood pressure monitoring and focused cardiac exam (cardiotoxicity), because for some patients these were triggered by other radiation exposures lacking dose‐specific thresholds.

**TABLE 3:** Number of recommended screening practices for organs with radiation dose‐specific thresholds^a^

	Irradiated regions		Volumetric dosimetry			
	At risk^b^	Tests over remainder of lifetime^c^	At risk^b^	Tests over remainder of lifetime^c^	Absolute Difference	% Difference
Hypothalamic pituitary axis						
Central adrenal insufficiency						
Medical Alert bracelet^d^	78	5472	49	3367	−2105	−38.5%
8 AM cortisol level	73	5135	49	3367	−1768	−34.4%
Steroid stress dosing	73	5135	49	3367	−1768	−34.4%
Focused endocrine history^d^	99	6898	95	6590	−308	−4.5%
Cochlea						
Ototoxicity						
Otic history and exam	73	5135	34	2385	−2750	−53.6%
Audiogram	73	1126	34	524	−602	−53.5%
Mandible						
Osteoradionecrosis						
Jaw history and exam	63	4441	1	69	−4372	−98.4%
Heart						
Cardiotoxicity						
Focused cardiac history	52	3597	16	1104	−2493	−69.3%
Cardiac health counseling	52	3597	16	1104	−2493	−69.3%
EKG	52	52	16	16	−36	−69.2%
Echocardiogram	52	1594	16	530	−1064	−66.8%
Cardiac exam^d^	118	8259	54	3772	−4487	−54.3%
Blood pressure^d^	55	3820	47	3262	−558	−14.6%
Spleen						
Functional asplenia						
Infectious risk counseling	5	337	0	0	−337	−100%
Medical Alert bracelet^d^	78	5472	49	3367	−2105	−38.5%

^a^ As designated by COG guidelines, anatomic structures with specific dose thresholds that must be surpassed to trigger late effect surveillance

^b^ Number of patients flagged for surveillance using this recommended screening practice

^c^ Total number of tests recommended to be done for full cohort over a remaining lifespan up to 80 years of age per patient

^d^ Recommended screening practices also triggered by potential late effects that have no dose‐specific thresholds

## 4 DISCUSSION

In this study, we sought to determine whether the use of VD rather than IR for identifying irradiated organs at risk might have potential for refining radiation‐related late effects screening as part of applying established guidelines for survivorship care. Consistent with our hypothesis, use of VD significantly reduced the number of potential late effects identified and corresponding recommended screening practices. This reduction was noted across all categories of screening practices and was most pronounced for organs or anatomic structures where a radiation dose threshold was specified by COG guidelines. Equally striking was our finding that additional organ screening was triggered in one‐third of our cohort by using VD. These findings are impactful because they suggest more accurate determination of radiation exposure can increase the yield of guideline‐driven care through enhanced surveillance for appropriate patients but decrease the cost and burden of unnecessary screening for most. To our knowledge, this study is the first to examine quantitatively the effect of using VD in guideline‐driven follow‐up care of long‐term childhood cancer survivors. Our efforts are consistent with recent refinements of the COG guidelines for reduced monitoring of survivors with CBCs, urinalyses, and echocardiograms.^18^, ^19^

The substantial difference in anatomic structures triggered for surveillance when using IR and VD reflects the relative inexactness of guideline defined radiation fields. Complete agreement of the two methods was rare: only 8 (6.1%) patients had the same organ‐specific late effects and recommended screening practices identified. While use of VD flagged significantly fewer potential late effects overall, it is notable that VD triggered additional surveillance for 30.3% of patients, indicating that IR may underestimate or misidentify organ‐specific exposure. These findings, along with the remarkable discordance between methods, imply that VD offers greater precision for determining late effect risk.

The significantly reduced mean number of recommended screening practices using VD has potential for increasing the low yield of some late effects surveillance practices.^18^ Given that full adherence to recommendations has been historically poor, minimizing unnecessary surveillance could improve the ability and willingness of survivors to follow recommendations.^20^, ^21^, ^22^ In this respect, the category of *diagnostic imaging and procedures* is notable because it comprises colonoscopies, mammograms, and echocardiograms. The nearly 40% reduction in recommendations with VD in this category may be especially impactful because these tests entail greater invasiveness, medical risk, higher cost, potential for false positive findings and subsequent workup, and associated “scanxiety.”^23^

A striking finding was the impressive number of recommended screening practices triggered by either method, with means ranging from 200 to almost 750 unique interventions per patient per decade of follow‐up. Most recommendations represent history and physical examination elements. While many of those are relatively benign, such as focused ophthalmologic history or limb length measurement, some are more intrusive and potentially distressing, e.g., detailed sexual history, breast examination, and Tanner staging.

The effect of VD was most dramatic for anatomic structures where COG guidelines indicate a dose‐specific threshold for risk. Currently, these are relatively few. However, organ‐specific radiation dose–volume thresholds have been determined for every major organ system via QUANTEC (Quantitative Analysis of Normal Tissue Effects in the Clinic) for adults,^24^ and a similar, ongoing initiative for children called PENTEC (Pediatric Normal Tissue Effects in the Clinic).^10^, ^11^ With these on the horizon, our results suggest that increased future use of VD could be impactful on a larger scale.

However, even for organs without specified dose thresholds, VD flagged significantly fewer potential late effects. That this effect was not even greater is probably due to the very conservative threshold we used for considering these organs substantially exposed (10% organ dose of 5 Gy). Under this assumption, some anatomic structures were classified at‐risk though the actual exposure was probably insignificant. This scenario was common for patients who received craniospinal irradiation, where VD frequently triggered screening in *laboratory tests* for lung, liver, and pancreas‐related late effects, while IR did not.

Implementation of this methodology on a larger scale will require multi‐institutional studies to compare IR and VD. We recognize that at some institutions, pediatric, radiation oncology, and survivorship patient care may not be fully integrated. Comprehensive treatment profiles personalized for each patient, often referred to as survivorship care plans,^25^ allow for integration of this data, and can be compiled by the specific treating providers at the end of therapy. Approaches have been developed to automate the extraction of this information, facilitating the creation of patient‐specific guides.^26^ Such care plans have been shown to enhance survivors' understanding of their follow‐up needs ^27^ and create a crucial reference for future care providers.^28^ Use of VD to determine late effects surveillance aligns with the goal to improve patient knowledge of their own health state while taking advantage of available technology and personalized medicine.

This study has several strengths and some limitations. Inclusion of a variety of diagnoses and irradiated body regions is a significant strength, making the cohort representative and our results generalizable. However, despite the diversity of our cohort, we recognize that all patients are drawn from a database of long‐term survivors, potentially introducing bias. Additional strengths include patients serving as their own controls to minimize bias and error and use of automated software for compiling volumetric dosimetry. This also indicates the feasibility and scalability of the approach. The use of real patient data, including unique anatomy and radiation treatment plans, is another strength of this work, supporting its real‐world implications. Limitations include our exclusive focus on radiation‐related late effects and surveillance, which, by only including anthracyclines, could underestimate the total burden of recommended surveillance. However, we felt that our approach was appropriate for this proof‐of‐concept study. Additionally, while contouring can be inexact, particularly with small volume structures, we incorporated automation and internal review to minimize this. Finally, we did not account for comprehensive dose–volume data, such as maximum dose received, however, this will be addressed with future incorporation of PENTEC guidelines.

What implications follow from this study? First, it should be emphasized these results reflect the impact of using accurate, organ‐specific radiation dose exposure when applying the COG guidelines, not a change in the guidelines themselves. Second, although survivors treated with both irradiation and chemotherapy might also be impacted by using VD, a separate cohort study is needed to establish this. Finally, reduced screening could have impacts on other outcome measures, including cost, time, and emotional burden for survivors, as well as minimizing morbidities from unnecessary interventions. Demonstrating benefits in these metrics would achieve an important goal of “precision survivorship,”^13^, ^29^ and should be explored with additional studies.

## 5 CONFLICT OF INTEREST

None.

## Supporting information

Supplementary Material

## Data Availability

Data available on request from the authors.
