# Supplementary material for: Surveillance for radiation‐related late effects in childhood cancer survivors: The impact of using volumetric dosimetry
Source: Cancer Med. 2020 Dec 16;10(3):905–13. doi: 10.1002/cam4.3671 (PMC7897961; doi:10.1002/cam4.3671)
Supplement: Supplementary file 1 — Supplementary Material [file CAM4-10-905-s001.pdf]

Appendix Table A1: Organs exposed to radiation and associated potential late effects*

Organ/Anatomic Structure	Potential Late Effect
Bladder	Bladder malignancy
Bones	Musculoskeletal growth problems
Bones	Radiation induced fracture
Bowel space	Bowel obstruction
Bowel space	Chronic enterocolitis, fistula, strictures
Brain	Brain tumor (benign or malignant)
Brain	Clinical leukoencephalopathy
Brain	Neurocognitive deficits
Breasts	Breast cancer
Breasts	Breast tissue hypoplasia
Carotid arteries	Carotid artery disease
Cerebral vasculature	Cerebrovascular complications
Cochlea	Ototoxicity
Colon	Colorectal cancer
Esophagus	Esophageal stricture
Face	Craniofacial abnormalities
Frontal, ethmoid, sphenoid, and maxillary sinuses	Chronic sinusitis
Heart	Cardiac toxicity
Hypothalamus, pituitary	Central adrenal insufficiency
Hypothalamus, pituitary	Central hypothyroidism
Hypothalamus, pituitary	Gonadotropin deficiency
Hypothalamus, pituitary	Growth hormone deficiency
Hypothalamus, pituitary	Hyperprolactinemia
Hypothalamus, pituitary	Overweight/obesity
Hypothalamus, pituitary	Precocious puberty
Kidney	Renal toxicity, insufficiency, hypertension
Lens	Cataracts
Liver	Cholelithiasis
Liver	Dyslipidemia
Liver	Hepatic fibrosis, cirrhosis, focal nodular hyperplasia
Lungs	Lung cancer
Lungs	Pulmonary toxicity
Mandible	Osteoradionecrosis of the jaw
Oral cavity	Dental abnormalities, temporomandibular joint dysfunction

Appendix Table A1, continued

Orbit, optic nerve	Ocular toxicity
Ovaries	Ovarian hormone deficiencies
Ovaries	Reduced ovarian follicular pool
Pancreas	Impaired glucose metabolism
Parotid, submandibular glands	Xerostomia, salivary gland dysfunction
Skin	Dermatologic toxicity
Skin, soft tissue, bone	Secondary benign or malignant neoplasm
Spine	Scoliosis/kyphosis male
Spleen	Functional asplenia
Subclavian arteries	Subclavian artery disease
Testes	Impaired spermatogenesis
Testes	Testicular hormonal dysfunction
Thyroid	Hyperthyroidism
Thyroid	Hypothyroidism
Thyroid	Thyroid cancer
Thyroid	Thyroid nodules
Ureter, bladder	Urinary tract toxicity
Uterus	Uterine vascular insufficiency
Vagina	Vaginal fibrosis/stenosis

*As outlined in the Children's Oncology Group (COG) Long-Term Follow-Up Guidelines for Survivors of Childhood, Adolescent, and Young Adult Cancer, version 5.0.[7]

Appendix Table A2: Recommended screening practices by category

Recommended Screening Practice*	Category^
Breast MRI	Diagnostic Imaging and Procedures
Colonoscopy	Diagnostic Imaging and Procedures
Complete audiological evaluation	Diagnostic Imaging and Procedures
Echocardiogram	Diagnostic Imaging and Procedures
EKG	Diagnostic Imaging and Procedures
Mammogram	Diagnostic Imaging and Procedures
Pulmonary functions testing	Diagnostic Imaging and Procedures
Assess thyroid levels prior to attempting pregnancy and throughout	Health Education and Counseling
Avoid frequent contact with irritants	Health Education and Counseling
Avoid obesity and management of obesity-related health risks	Health Education and Counseling
Avoid tobacco and quit smoking if appropriate	Health Education and Counseling
Be aware of adverse impact of hormone deficiencies on growth, BMD, CV disease, and sexual dysfunction	Health Education and Counseling
Be aware of increased risk of fractures	Health Education and Counseling
Be aware of need for corticosteroid replacement therapy and stress dosing	Health Education and Counseling
Be aware of potential for shorter period of fertility	Health Education and Counseling
Be aware of risk of life threatening and malarial and tick-borne disease	Health Education and Counseling
Be aware recovery of fertility may occur years later (female)	Health Education and Counseling
Be aware spermatogenesis may occur years later (male)	Health Education and Counseling
Control health conditions known to increase cardiovascular and stroke risk	Health Education and Counseling
Exercise safety counseling as appropriate	Health Education and Counseling
Maintain appropriate weight, blood pressure, heart healthy diet	Health Education and Counseling
Possibility of need for spiral CT with any suspicion for lung pathology	Health Education and Counseling
Promptly report dysuria or gross hematuria	Health Education and Counseling
Promptly seek medical attention for bone pain, bone mass, persistent fevers	Health Education and Counseling
Teach breast self-exam and counsel to perform monthly	Health Education and Counseling

Appendix Table A2, continued

Use contraception regularly	Health Education and Counseling
Wear Medical Alert bracelet	Health Education and Counseling
Assessment of bone pain and health	History and Physical Exam Element
Assessment of nutritional status	History and Physical Exam Element
Bilateral upper extremity blood pressure monitoring	History and Physical Exam Element
Blood pressure monitoring	History and Physical Exam Element
Brachial and radial pulses assessment	History and Physical Exam Element
Breast exam	History and Physical Exam Element
Carotid exam	History and Physical Exam Element
Comprehensive jaw exam	History and Physical Exam Element
Comprehensive jaw history	History and Physical Exam Element
Craniofacial exam	History and Physical Exam Element
Detailed pubertal history	History and Physical Exam Element
External genitalia exam	History and Physical Exam Element
Eye exam	History and Physical Exam Element
Focused abdominal exam	History and Physical Exam Element
Focused cardiac exam	History and Physical Exam Element
Focused cardiac history	History and Physical Exam Element
Focused dermatologic exam	History and Physical Exam Element
Focused dermatologic history	History and Physical Exam Element
Focused educational history	History and Physical Exam Element
Focused endocrine history	History and Physical Exam Element
Focused exam for dermatologic evidence of endocrine disorder	History and Physical Exam Element
Focused gastrointestinal history	History and Physical Exam Element
Focused genitourinary history	History and Physical Exam Element
Focused hair exam	History and Physical Exam Element
Focused hepatic exam	History and Physical Exam Element
Focused neurologic exam	History and Physical Exam Element
Focused neurologic history	History and Physical Exam Element
Focused ophthalmologic symptomatology assessment	History and Physical Exam Element
Focused oral and dental exam	History and Physical Exam Element
Focused otic history	History and Physical Exam Element
Focused pulmonary history	History and Physical Exam Element
Focused sinus exam	History and Physical Exam Element
Focused sinus history	History and Physical Exam Element

Appendix Table A2, continued

Focused urologic history	History and Physical Exam Element
Focused vision history	History and Physical Exam Element
Height and weight	History and Physical Exam Element
Height/Weight/BMI	History and Physical Exam Element
History of galactorrhea	History and Physical Exam Element
Limb lengths	History and Physical Exam Element
Menopausal symptoms	History and Physical Exam Element
Menstrual and pregnancy history	History and Physical Exam Element
Menstrual history	History and Physical Exam Element
Otoscopic exam	History and Physical Exam Element
Palpation of bones in irradiated fields	History and Physical Exam Element
Perfusion exam	History and Physical Exam Element
Pregnancy history	History and Physical Exam Element
Psychosocial assessment	History and Physical Exam Element
Pulmonary exam	History and Physical Exam Element
Regular growth monitoring	History and Physical Exam Element
Sexual function history	History and Physical Exam Element
Sitting height	History and Physical Exam Element
Skin self-exam	History and Physical Exam Element
Spine exam	History and Physical Exam Element
Tanner staging	History and Physical Exam Element
Testicular volume by Prader orchidometry	History and Physical Exam Element
Thyroid exam	History and Physical Exam Element
8 AM Cortisol level	Laboratory Test
Electrolyte panel	Laboratory Test
Fasting blood glucose or HbA1c	Laboratory Test
Lipid profile	Laboratory Test
Liver function testing	Laboratory Test
Thyroid function tests	Laboratory Test
Referral for dental exam and cleaning	Referral to Specialist
Referral for formal neuropsychological evaluation	Referral to Specialist
Referral for ophthalmology evaluation	Referral to Specialist

*As outlined in the Children's Oncology Group (COG) Long-Term Follow-Up Guidelines for Survivors of Childhood, Adolescent, and Young Adult Cancer, version 5.0.[7]

^As defined in this study.
